# Risk Indicators and Treatment Needs of Children 2–5 Years of Age Receiving Dental Treatment under General Anesthesia in Saskatchewan

**DOI:** 10.3390/dj10010008

**Published:** 2022-01-06

**Authors:** Alyssa Weninger, Erica Seebach, Jordyn Broz, Carol Nagle, Jessica Lieffers, Petros Papagerakis, Keith Da Silva

**Affiliations:** 1 College of Dentistry, University of Saskatchewan, Saskatoon, SK S7N 5E4, Canada; alyssa.weninger@usask.ca (A.W.); erica.seebach@usask.ca (E.S.); jordyn.broz@usask.ca (J.B.); cl.nagle@usask.ca (C.N.); petros.papagerakis@usask.ca (P.P.); 2 College of Pharmacy and Nutrition, University of Saskatchewan, Saskatoon, SK S7N 5E5, Canada; jrl210@mail.usask.ca

**Keywords:** dental caries, anesthesia, general, pediatric dentistry, cross-sectional studies, caregivers

## Abstract

Background: When compared to national averages in Canada, Saskatchewan has one of the highest rates of dental treatment under general anesthesia (GA) and average costs per child. Thus, the purpose of this cross-sectional study is to explore the risk indicators and treatment needs of children receiving dental treatment under GA in Saskatchewan. Methods: In this cross-sectional study, we recruited caregivers of children between 24 and 71 months of age in Saskatoon, Canada. Caregivers completed a 40-item questionnaire, which was supplemented with clinical data and then subject to statistical analysis (independent t-tests and one-way ANOVA). Results: A total of 90 caregiver/child dyads were enrolled with the mean age for children being 49.5 ± 12.3 months. The mean age of a child’s first dental visit was 34.7 ± 15.3 months with only 37.9% of children having a dental home. The mean deft index was 11.7 ± 3.4, with an average of 10.9 ± 3.5 teeth receiving treatment. Additionally, location of primary residence (*p* = 0.03), family income (*p* = 0.04), family size (*p* = 0.01), parental education (*p* = 0.03), dental home (*p* = 0.04), and body mass index (*p* = 0.04) had a statistically significant association with a higher mean deft. Conclusions: Our cross-sectional study confirms that children who require dental treatment under GA have a high burden of disease. While individual risk indicators such as diet and oral hygiene play a role in the progression of early childhood caries (ECC), we also demonstrate that children who do not have access to early preventive visits or a dental home are at a higher risk. In addition to improving motivation for oral hygiene at home and nutritional education, improving access to oral health care should be addressed in strategies to reduce ECC.

## 1. Introduction

Dental caries is one of the most prevalent diseases worldwide, affecting nearly two-thirds of all Canadian children aged 6 to 11 [1,2]. Furthermore, children from low socio-economic groups are particularly vulnerable to the disease [3]. Early childhood caries (ECC) is defined as the presence of one or more decayed, missing (due to caries), or filled tooth surfaces in any primary tooth in a child under the age of six [4]. It can be further classified as being severe (S-ECC) when any smooth surface caries are found on a child under the age of three [4]. While the primary etiology of ECC involves the activity of cariogenic bacteria over time, the list of factors that may increase ECC risk is long. There are individual factors such as plaque microbiology, developmental defects, saliva composition, chronic disease, genetics, and previous caries experience [5,6]. There are behavioural and environmental factors such as diet, which include the frequency of fermentable carbohydrate intake and bottle-feeding habits, oral hygiene, fluoride exposure, and exposure to other non-fluoride preventive interventions such as amorphous calcium phosphate and substituted zinc biomimetic hydroxyapatite [5,6,7]. Beyond individual-level factors, there are also familial and socioeconomic risk indicators to consider such as caregiver caries experience, education level, socioeconomic status, insurance coverage, and the ability to access dental care [5,6]. These factors encompass the social determinants of health, or the conditions in which people are born, live, and work that can affect their health [8]. When left untreated, ECC leads to infection, pain, and premature loss of teeth and can have deleterious effects over time with children ultimately suffering from a reduced oral health related quality of life [9,10,11].

While there are minimally invasive approaches to managing ECC [12], treatment of advanced disease requires a surgical approach to restore or remove teeth that are beyond repair. Due to the extensive nature of the disease and often the maturity and reactive behaviour of young children, delivery of treatment can be challenging for dentists. Thus, comprehensive dental treatment under general anesthesia (GA) has become a common approach. In Canada, Schroth et al. report that the overall rate of outpatient surgery under GA to treat ECC was 12.1 per 1000 under the age of 5, accounting for 31% of all day surgeries performed in this age group [13]. Although dental treatment for children under GA is considered safe and effective, there is still concern with the high rates of caries recurrence and re-treatment [14,15]. Further, an association between early exposure to GA and neurotoxicity has been established in pre-clinical studies and, clinically, there is some evidence that multiple exposures to GA before age 3 are associated with decreased processing speed and fine motor abilities, and increased deficits in reading and behaviour [16,17,18]. As such, there is a need to further explore the factors that may increase the risk of dental treatment under GA with the aim of developing more effective preventive strategies.

When compared to national averages in Canada, Saskatchewan has one of the highest rates of dental treatment under GA and average costs per child [13]. To date, there have been no studies that have explored dental treatment under GA in Saskatchewan in children 2–5 years of age. Without more information, it is difficult to assess the extent of this treatment, as well as whether this problem may be exacerbated by certain risk factors. Thus, the purpose of this cross-sectional study is to explore the patterns and risk indicators associated with dental treatment under GA in Saskatchewan. We hypothesize that children requiring GA for dental treatment will present with a high burden of disease and will be affected by social determinants of health.

## 2. Materials and Methods

This exploratory cross-sectional study was approved by the University of Saskatchewan Biomedical Research Ethics Board (BioID 74746). A convenience sample of caregivers of children between 24 and 71 months of age attending the Prairieview Surgical Center (PSC) for dental treatment between June and August 2018 were recruited to participate. PSC is a private GA facility located in Saskatoon, Saskatchewan, and only children who are classified as ASA II or below, (American Society of Anesthesiologists Physical Status Classification System) are admitted. Based on the volume of children treated at PSC, a confidence interval of 95%, and a 5% margin of error, we determined our minimum sample size to be 80. Caregivers were guided through an informed consent process and those who agreed to participate were asked to complete an electronic questionnaire via SurveyMonkey^®^ software (SurveyMonkey Inc., San Mateo, CA, USA). The questionnaire was developed based on a review of the literature and the research team’s a priori knowledge of the subject, and was pilot-tested and refined prior to distribution. The 40-item questionnaire captured demographic information, dietary habits, oral hygiene behaviours, dental care utilization, and other potential risk factors or behaviours known to be associated with caries. In regard to the child’s place of residence, postal codes were used to determine if the child lived within the Saskatoon city limits, within a 2 h driving commute to Saskatoon, or greater than a 2 h driving commute to Saskatoon. The variable for parental education was dichotomized to the categories ‘high school and below’ (if the highest level of education attainted was a high school diploma) and ‘above high school’ (if respondents had received a college certificate/diploma, undergraduate degree, graduate degree or higher).

Following completion of the dental treatment under GA, the principal dentist submitted their clinical findings and a list of all treatment provided on a standardized form. The ‘deft index’ was determined for each child. This index represents the total count of all the primary teeth that were decayed, missing to due extraction, or filled based on the World Health Organization (WHO) criteria for dental surveys [19]. Each child’s body mass index (BMI) was calculated using the height and weight measured on the day of surgery. The BMI was then adjusted for age and converted to percentiles based off guidelines of the WHO Growth Charts for Canada [18]. Children were considered to above normal weight if their BMI-for-age was ranked at or above the 85th percentile.

Data analysis began with descriptive and summary statistics; continuous variables are presented as mean ± standard deviation (SD). Differences in means were determined using independent *t*-tests and one-way ANOVA’s followed by a Tukey’s HSD post hoc test where appropriate. A *p*-value of 0.05 was used to determine statistical significance. All analyses were performed using IBM SPSS Statistics (version 25.0, IBM Corp, Armonk, NY, USA).

## 3. Results

A total of 90 caregiver/children dyads completed the study protocol representing a response rate of 85.7%. Participant demographics are presented in Table 1. The mean age for children included in the study was 49.5 ± 12.3 months. For principal residence, 45.6% of the children lived within the Saskatoon city limits, 34.4% lived within a 2 h commute, and 20.0% of participants lived greater than a 2 h commute from Saskatoon. Considering socioeconomic indicators, 52.2% of caregiver participants reported high school as their highest level of education, and 57.5% reported a total household income less than USD 50,000. While 86.7% of the participants reported having dental insurance, it was unknown whether the coverage was from public or private sources.

A summary of findings regarding ECC risk indicators is presented in Table 2. The mean age of the first dental visit was 34.7 ± 15.3 months and only 37.9% of children had established a consistent dental home. For 91.0% of children, this was their first time receiving dental treatment under GA. In addition, 48.7% of children had at least one sibling who had previously required treatment under GA. When looking at self-reported oral hygiene behaviours, 73.3% of children were brushing their teeth at least once per day, and 69.7% caregivers reported that they assisted their child with brushing. The majority of children (70.1%) were breastfed; however, 78.9% of the sample also used bottles, with 76.7% of these caregivers reporting that the children also routinely went to sleep with a bottle. Caregivers also reported that their children were snacking on average 3.4 ± 1.5 times/day on top of regular meals.

The clinical findings and dental treatment completed as reported by the principal dentist is summarized in Table 3. With respect to BMI, 41.6% of children were considered to be above normal weight as measured by percentile ranks. Almost all children has signs of visible debris and/or plaque (95.6%). The degree of caries severity for the sample was high, with a mean deft index of 11.7 ± 3.4, and with an average of 10.9 ± 3.5 teeth receiving treatment on the day of surgery. Children required a variety of different procedures across a gradient of severity. On average per child, 1.9 ± 2.1 teeth required restorations, and 6.9 ± 2.7 teeth required crowns. In addition, on average per child, 1.8 ± 1.7 teeth required vital pulp therapy and 2.0 ± 2.7 teeth required extractions.

The association of known risk indicators and high deft scores is summarized in Table 4. On average, children living further than 2 h away from Saskatoon had higher deft index scores when compared to children who lived within city limits, or less than 2 h away, respectively (13.6 ± 3.2 vs. 11.6 ± 3.5 and 10.8 ± 3.1: *p* = 0.03). There was no significant difference in deft scores for children who lived within city limits versus those who lived within 2 h away. Children from households with greater than five individuals also had significantly higher deft index scores compared to those who had less than five individuals (12.7 ± 3.3 vs. 10.8 ± 3.4; *p* = 0.01). Additionally, significantly higher deft index scores were noted for families whose total household income was less than USD 50,000 compared to those with an income of USD 50,000 or more (12.2 ± 3.1 vs. 10.7 ± 3.2; *p* = 0.04) or when high school was the highest caregiver level of education attained compared to those with greater than a high school education (12.5 ± 3.5 vs. 10.9 ± 3.2; *p* = 0.03). Children who did not have a dental home had significantly higher deft scores compared to those who did (12.8 ± 10.2 vs. 10.2 ± 1.5; *p* = 0.03) as did children who were above normal weight as measured by BMI compared to those who were not (12.7 ± 3.5 vs. 11.1 ± 3.3; *p* = 0.04). No significant differences in deft index scores were found when considering dental insurance, caregiver caries experience, sibling GA history, bottle feeding when going to sleep, or reported oral hygiene behaviour.

## 4. Discussion

This study explores the individual and socioeconomic risk indicators of children who require extensive treatment under GA in Saskatoon, Saskatchewan. Consistent with existing literature, we confirm that children who require dental treatment under GA have a high burden of dental disease and extensive treatment needs [13,14]. Additionally, our proxy indicators for social determinants of health, such as location of residence, income, education, and utilization of dental care services, were associated with higher deft scores. Understanding these risk indicators is essential when considering clinical and community interventions to better serve children at risk for ECC. Considering both the public and private costs of dental treatment under GA, as well as the additional risks that may come with exposure to GA at an early age, it is incumbent on oral healthcare professionals to explore any and all interventions aimed at decreasing these rates.

Consistent with other studies, children in our sample population exhibited many common individual and behavioural risk factors for ECC [5,20,21]. These include going to sleep with a bottle, a high frequency of snacking, and insufficient oral hygiene. Oral health promotion and educational interventions at both the clinical and community level in Saskatchewan, such as targeted nutritional counselling to high risk populations, have targeted many of these individual factors. However, one challenge is that children and their families may not be exposed to this information until they are in the school-system, which may already be too late. Additionally, it has been shown that oral health education alone may not be sufficient to have a population level effect on caries reduction and more robust interventions may be indicated [22]. Finding ways to reach high-risk children and their families at an earlier age is paramount to the success of any preventive program.

It is well documented that early dental visits aimed at risk assessment and prevention can be effective at improving oral health-related outcomes and costs [23,24,25]. Children who visit the dentist early often have fewer treatment needs compared to those children who only start seeing a dentist in their mixed dentition stage [24]. It has, therefore, been recommended that children should have their first exam by the age of one [26]. Further, children should have access to a dental home, where all aspects of their oral health care can be coordinated and delivered in a comprehensive, continuously accessible, and family-centered way [26,27]. With the ability to detect caries in its earliest stages, oral healthcare professionals can help arrest the progression of disease, prevent cavitation, and even reverse damage to the teeth in some cases, all before surgical intervention is necessary [28,29,30,31]. Thus, it is possible to decrease the need for more extensive treatment under GA. In our sample, it was found that children who required GA did not have their first dental visit until they were approximately three years old, and only 37.9% of children had established a dental home. Additionally, children who did not have a dental home had a significantly higher deft index scores. A greater emphasis on early detection of dental caries and the year one visit is required, which will include a better understanding of Canadian dentists’ compliance with the recommendations for the age one dental visit which has been previously shown as an area of concern [32].

Timely access to oral health care remains a challenge across Canada and Internationally. Many children from low-income and/or uninsured families, as well as those who live in Northern and more remote areas suffer disproportionately from the adverse effects of poor oral health [3]. In our sample, indicators for the social determinants of health including lower household incomes, caregiver education, location of residence, and utilization of dental care services were all associated with a higher deft index score. We also found the children in our sample who were above a normal BMI range or obese, another chronic condition associated with the social determinants of health, had higher deft index scores. Addressing the social determinants of health is based on the principle that improving social conditions is essential for improving health outcomes for vulnerable populations, reducing inequalities, and improving health equity [8]. Previous research has demonstrated gradients in oral health outcomes based on socioeconomic position consistent with our findings [33]. More recently, a biological link between socioeconomic factors, stress hormones and increased cariogenic bacteria counts was proposed [34]. This again emphasizes the importance of addressing the social determinants of oral health as a part of any comprehensive intervention to reduce the burden of ECC and subsequent use of GA for treatment.

This study has several limitations that must be taken into consideration. With a cross-sectional design, we are unable to make inferences about causation of ECC or treatment under GA. Additionally, this questionnaire has not been validated, and data emerging from caregiver’s responses is subject to a degree of recall bias. Finally, due to the relatively small sample size and the location of the study, caution must be taken when generalizing these findings.

## 5. Conclusions

Children who bear the greatest burden of ECC are usually those with the greatest difficulty accessing oral health care services. The purpose of this cross-sectional study is to explore the patterns and risk indicators associated with dental treatment under GA in Saskatchewan. Our study confirms our hypothesis that children who are receiving dental treatment for ECC under GA in Saskatoon, Saskatchewan have a high burden of disease. While individual risk factors such as diet and oral hygiene play a role in the progression of ECC, we also demonstrate that children with a higher burden of the disease may not have access to early preventive visits or a dental home. Social determinants of health were also associated with poor oral health status. To be able to reduce the burden of disease for vulnerable children, and thus rates of GA use in Saskatchewan, oral health education and health promotion alone may not be sufficient. A collaborative and interdisciplinary approach that combines clinical and community-based interventions while addressing the social determinants of health is likely required.

## Figures and Tables

**Table 1:** Sample population demographics (*n* = 90).

Variable	*n* (%) or Mean ± Standard Deviation
Child age (months)	49.5 ± 12.3
Child sex (% female)	44 (48.9)
Location of residence	
Within Saskatoon	41 (45.6)
Within a 2-h commute	31 (34.4)
Greater than a 2 h commute	18 (20.0)
Parental education	
High school or below	47 (52.2)
Above high school	43 (47.8)
Household income	
Less than USD 50,000	46 (57.5)
USD 50,000 or more	34 (42.5)
Number of people living in household (mean)	5.4 ± 2.4
Number of people living in household (proportion)	
5 or less	54 (60.7)
Greater than 5	35 (38.9)
Child has at least 1 sibling	77 (86.5)
Dental insurance (% yes)	78 (86.7)
Smoking in household (% yes)	64 (71.1)
Access to fluoridated tap water (% yes)	37 (46.3)

**Table 2:** Risk indicators for early childhood caries.

Variable	*n* (%) or Mean ± Standard Deviation
Age of first dental visit (months)	34.7 ± 15.3
Dental home (% yes)	33 (37.9)
First dental treatment under GA (% yes)	81 (91.0)
Sibling required treatment under GA (% yes)	37 (48.7)
Parental positive caries experience (% yes)	80 (88.9)
Frequency of tooth brushing	
Less than once per day	32 (26.7)
At least one per day	63 (73.3)
Who brushes child’s teeth	
Child only	27 (30.3)
Parental assistance	62 (69.7)
Breastfed (% yes)	61 (70.1)
Weaning age (months)	10.7 ± 8.7
Bottle-fed (%yes)	71 (78.9)
Bottle to sleep (% yes)	56 (76.7)
Weaning age (months)	23.2 ± 8.5
Meals per day	2.9 ± 0.6
Snacks per day	3.4 ± 1.5
Access to fluoridated tap water (% yes)	37 (46.3)

GA: general anesthesia.

**Table 3:** Summary of clinical findings and dental treatment.

Variable	*n* (%) or Mean ± Standard Deviation
BMI	16.6 ± 2.11
Weight group	
Normal weight or below	52 (58.4)
Above normal weight	37 (41.6)
Visible plaque (% yes)	86 (95.6)
Visible enamel hypoplasia (% yes)	9 (10.1)
Deft index	11.7 ± 3.4
Total teeth treated	10.9 ± 3.5
Total restorations	1.9 ± 2.10
Anterior restorationsNormal weight or below	1.3 ± 1.7
Posterior restorationsAbove normal weight	0.7 ± 1.3
Total crowns	6.9 ± 2.7
Anterior resin crowns	0.8 ± 1.4
Posterior stainless steel crowns	6.1 ± 2.3
Total pulp treatments	1.8 ± 1.7
Total extractions	2.0 ± 2.7
Anterior extractions	1.5 ± 2.1
Posterior extractions	0.5 ± 1.1
Access to fluoridated tap water (% yes)	37 (46.3)

BMI: Body Mass Index; deft: decayed, extracted, and filled teeth index.

**Table 4:** Differences in mean deft indices.

Variable	Deft Mean ± Standard Deviation	*p*-Value
Location of residence		0.03 ^β^
Within Saskatoon	11.6 ± 3.5	
Within a 2 h commute	10.8 ± 3.1	
Greater than a 2 h commute	13.6 ± 3.2	
Parental education		0.03 ^α^
High School or below	12.5 ± 3.5	
Above high school	10.9 ± 3.2	
Household income		0.04 ^α^
<USD 50,000	12.2 ± 3.1	
USD 50,000 or more	10.7 ± 3.2	
People in household		0.01 ^α^
5 or less	10.8 ± 3.4	
Greater than 5	12.7 ± 3.3	
Dental Insurance		0.80 ^α^
Yes	11.5 ± 3.5	
No	11.7 ± 3.1	
Dental home		0.04 ^α^
Yes	10.2 ± 1.5	
No	12.8 ± 1.1	
Weight group		0.04 ^α^
Normal weight or below	11.1 ± 3.3	
Above normal weight	12.7 ± 3.5	

^α^ Independent *T*-Test; ^β^ one-way ANOVA.

## Data Availability

The data presented in this study are available on request from the corresponding author. The data are not publicly available due to privacy.
